# Ishak stage 6 fibrosis is more likely to regress than stage 5 in CHB patients undergoing entecavir therapy

**DOI:** 10.3389/fmed.2026.1765594

**Published:** 2026-03-09

**Authors:** Mengjie Li, Xiaogang Hao, Yuanyuan Li, Chao Zhang, Yongping Chen, Zujiang Yu, Qin Li, Lin Tan, Dedong Xiang, Qinghua Shang, Chunliang Lei, Liang Chen, Xiaoyu Hu, Jing Wang, Huabao Liu, Wei Lu, Yan Chen, Zheng Dong, Wenlin Bai, Eric M. Yoshida, Nahum Mendez-Sanchez, Ke-Qin Hu, Xingshun Qi, Yongping Yang, Jingfeng Bi

**Affiliations:** 1 School of Pharmaceutical Sciences, Shandong University of Traditional Chinese Medicine, Jinan, China; 2 Department of Phase I Clinical Trial Ward, Senior Department of Infectious Diseases, Chinese PLA General Hospital, National Clinical Research Center for Infectious Diseases, Beijing, China; 3 Department of Inpatient and Medical Record Management, Chinese PLA General Hospital, Beijing, China; 4 Department of Infectious Diseases, Chinese PLA General Hospital, Beijing, China; 5 Department of Infectious and Liver Diseases, Liver Research Center, the First Affiliated Hospital of Wenzhou Medical University, Wenzhou, Zhejiang, China; 6 Department of Infectious Disease, the First Affiliated Hospital of Zhengzhou University, Zhengzhou, Henan, China; 7 Fuzhou Infectious Diseases Hospital, Fuzhou, Fujian, China; 8 Department of Liver Disease, Fuyang 2nd People’s Hospital, Fuyang, Anhui, China; 9 Department of Infectious Diseases, Southwest Hospital, the Third Military Medical University, Chongqing, China; 10 Therapeutic Center for Liver Disease, the 960th Hospital of PLA, Taian, Shandong, China; 11 Guangzhou 8th People’s Hospital, Guangzhou, Guangdong Province, China; 12 Department of Hepatic Diseases, Shanghai Public Health Clinical Center, Shanghai, China; 13 National Integrative Medicine Clinical Base for Infectious Diseases and Department of Infectious Diseases, Affiliated Hospital of Chengdu University of Traditional Chinese Medicine, Chengdu, Sichuan, China; 14 Affiliated Traditional Chinese Medicine Hospital of Southwest Medical University, Luzhou, Sichuan, China; 15 Traditional Chinese Medicine Hospital of Chongqing, Chongqing, China; 16 Tianjin Second People’s Hospital, Tianjin Institute of Hepatology, Tianjin, China; 17 Department of Liver Diseases, Chinese PLA General Hospital, Beijing, China; 18 Division of Gastroenterology, Vancouver General Hospital, Vancouver, BC, Canada; 19 Liver Research Unit, Medica Sur Clinic & Foundation, Mexico City, Mexico; 20 Faculty of Medicine, National Autonomous University of Mexico, Mexico City, Mexico; 21 Division of Gastroenterology and Hepatology, University of California, Irvine, School of Medicine, Irvine, CA, United States; 22 Department of Gastroenterology, General Hospital of Northern Theater Command (formerly General Hospital of Shenyang Military Area), Shenyang, Liaoning, China

**Keywords:** chronic hepatitis B, entecavir, fibrosis regression, Ishak stage, logistic regression

## Abstract

**Background:**

Chronic hepatitis B (CHB) patients with milder baseline fibrosis have traditionally been considered more likely to achieve histological improvement after antiviral therapy. However, our previous finding suggests that patients with Ishak stage 6 may have greater potential for fibrosis regression than those with stage 5. This study aimed to evaluate whether CHB patients with Ishak stage 6 are more likely to achieve fibrosis regression than those with stage 5 after entecavir (ETV) monotherapy or ETV in combination with Biejia-Ruangan (ETV + BR) therapy.

**Methods:**

Baseline Ishak fibrosis stage served as the main analytic variable, while the use of concomitant traditional Chinese medicine was included as a stratification factor. Demographic characteristics, viral markers, and baseline laboratory parameters were incorporated as covariates. A logistic regression model was applied to evaluate the association between baseline Ishak stage and fibrosis regression. Based on this model, a sensitivity analysis was further performed in the subgroup of patients with baseline Ishak stage 5 or 6 to assess the robustness of the findings. A generalized additive model (GAM) was additionally applied to explore potential non-linear stage-related patterns.

**Results:**

A total of 705 patients had paired biopsy data. The fibrosis regression rate was 50.21% in stage 6 versus 45.58% in stage 5. In the multivariable logistic regression analysis, baseline Ishak stage 6 was associated with a higher likelihood of fibrosis regression compared with stage 5 (*OR*:1.612, *95%CI*:1.027 ~ 2.529, *p* = 0.038). GAM analysis revealed a stage-related, non-linear trajectory with a nadir at stage 5 and an upward trend at stage 6. Sensitivity analyses yielded consistent results (*OR*: 1.835, *95%CI*: 1.148 ~ 2.931, *p* = 0.011).

**Conclusion:**

Among CHB patients treated with ETV, those with baseline Ishak stage 6 were more likely to achieve histological fibrosis regression than those with stage 5.

**Clinical trial registration:**

Identifier NCT01965418.

## 1 Introduction

Chronic hepatitis B virus (HBV) infection remains a major global public health challenge. The hepatitis B virus (HBV) is estimated to chronically infect approximately 254 million individuals worldwide, leading to around 1,100,000 liver disease–related deaths each year (1). Chronic hepatitis B (CHB) is characterized by persistent hepatic inflammation and progressive fibrosis, which can ultimately advance to cirrhosis, hepatic decompensation, and hepatocellular carcinoma (2).

The reversibility of liver fibrosis has been demonstrated in several studies, particularly among patients receiving long-term antiviral therapy at earlier stages of disease (3, 4). Entecavir (ETV), a first-line nucleoside analogue, has been extensively validated for its potent antiviral activity against HBV and its ability to improve liver histopathology (5, 6).

Histological staging remains the gold standard for fibrosis assessment, with the Ishak scoring system widely applied in clinical and research settings. In this system, stage 5 (early cirrhosis without complete nodule formation) and stage 6 (established cirrhosis) represent the terminal stages of fibrosis progression. Traditionally, earlier stages of fibrosis are considered more likely to regress with effective antiviral therapy (7). However, findings from our previous randomized controlled trial (RCT) (8) revealed a unexpected pattern—patients with baseline Ishak stage 6 exhibited a slightly higher rate of fibrosis regression compared with those at stage 5 (50.21% vs. 45.58%).

Given the potential clinical implications, the present study aimed to clarify the relationship between baseline Ishak fibrosis stage and histological regression in CHB patients receiving ETV-based therapy. Leveraging data from a large multicenter RCT, we systematically compared fibrosis regression rates between Ishak stage 5 and stage 6 patients using multivariable statistical modeling. Our goal was to generate robust evidence to inform individualized antiviral treatment strategies for CHB.

## 2 Methods

### 2.1 Data source

The data for this study were derived from a randomized, placebo-controlled, double-blind, multicenter clinical trial conducted between October 2013 and April 2016 (Clinical Trials Registration. NCT01965418) (8, 9). The trial was conducted in 14 institutions, including the Fifth Medical Center of the Chinese PLA General Hospital, and enrolled a total of 1,000 patients with chronic hepatitis B (CHB). Inclusion criteria were as follows: (1) age 18–65 years with chronic hepatitis B virus (HBV) infection and no history of prior antiviral therapy; (2) for HBeAg-positive CHB, HBV DNA ≥ 20,000 IU/mL and ALT ≥ 2 times the upper limit of normal (ULN); for HBeAg-negative CHB, HBV DNA ≥ 2,000 IU/mL and ALT ≥ 2 times the ULN; or clinically compensated cirrhosis, regardless of HBeAg status, with detectable serum HBV DNA at screening before liver biopsy, meeting at least one of the following criteria: (a) normal ALT and liver stiffness > 9 kPa, or elevated ALT < 5 times the ULN and liver stiffness > 12 kPa; (b) platelet count < 100,000/mL and ultrasonographic findings suggestive of cirrhosis, including a blunted or nodular liver edge accompanied by splenomegaly (spleen length > 12 cm); (c) clinical signs of portal hypertension, such as esophageal or gastric varices, in the absence of ascites, variceal hemorrhage, or hepatic encephalopathy; (3) liver histology showing an IFS ≥ 3; (4) naive to treatment with nucleos(t)ide analogues (NAs), or no HBV antiviral or antifibrotic therapy within at least 6 months prior to enrollment. Exclusion criteria included: (1) co-infection with other hepatotropic viruses; (2) concomitant non-viral liver diseases; (3) decompensated cirrhosis; (4) history of malignancy; (5) severe primary heart, kidney, or other major organ diseases, as well as psychiatric disorders; (6) pregnancy or lactation.

Eligible patients were randomized into two groups: the treatment group (*n* = 500) received entecavir plus the TCM compound Biejia-Ruangan, and the control group (*n* = 500) received entecavir plus placebo, both for a total of 72 weeks. All patients underwent liver biopsy before initiating antiviral therapy, and 705 patients underwent follow-up percutaneous liver biopsy under ultrasound guidance after 72 weeks of treatment. Among the 1,000 enrolled patients, 705 (70.5%) completed follow-up biopsy, which is comparable to rates reported in other studies requiring repeat liver biopsy. Overall, 500 patients were enrolled in each group as the intention-to-treat population. The rate of fibrosis regression after 72 weeks of treatment was significantly higher in the ETV + BR group (40% vs. 31.8%; *p* = 0.0069). Among 388 patients with cirrhosis (ie, IFS ≥ 5) at baseline, the rate of cirrhosis reversal (ie, IFS ≤ 4) was significantly higher in the ETV + BR group (41.5% vs. 30.7%; *p* = 0.0103).

Liver tissue samples were independently evaluated in a blinded manner by two pathologists, assessing both histological activity index (HAI) and fibrosis stage according to the Ishak scoring system. In cases of scoring discrepancy, the final fibrosis stage was determined by a third senior pathologist.

### 2.2 Inclusion criteria

Only patients who had complete liver histological data available at both baseline and at week 72 of treatment were included in the present analysis.

### 2.3 Data collection

Baseline variables included demographic data (age, sex, body mass index [BMI]); lifestyle factors (smoking and alcohol consumption); ultrasonographic features (spleen thickness and spleen length); and a wide range of laboratory parameters: hematologic indices (WBC, RBC, HGB, PLT, N%, MO, EO), liver-related enzymes (ALT, AST, ALP, GGT) and biochemical markers (TBIL, ALB, GLO), coagulation profiles (PT, APTT, TT, FIB), renal function markers (BUN, CR), virological markers (HBsAg, HBeAg, and HBV DNA), and alpha-fetoprotein (AFP).

### 2.4 Statistical analysis

Continuous variables were first categorized according to established clinical reference ranges. Patients were then divided into two groups based on the presence or absence of liver fibrosis regression. Baseline comparisons were performed for categorized continuous variables and other categorical variables, such as sex.

A multivariate logistic regression model was constructed with fibrosis regression as the dependent variable. The treatment regimen (entecavir alone or entecavir combined with Compound Biejia-Ruangan) was included as a stratification factor, and variables with *p* < 0.20 in the baseline analysis, together with HBV DNA levels (previously demonstrated to be an important determinant of fibrosis regression) (10), were incorporated as covariates for adjustment.

To further explore whether the relationship between baseline Ishak stage and fibrosis regression was non-linear, a generalized additive model (GAM) was applied. The model used thin plate regression splines as the smoothing function for Ishak stage to allow the effect to vary flexibly rather than assuming a straight-line relationship. The GAM included the same covariates as the logistic regression to ensure consistency and comparability between the two analyses.

Finally, a sensitivity analysis was performed in the subset of patients with baseline Ishak stage 5 or 6, using the same statistical approach to confirm the robustness of the findings. All statistical analyses were conducted using SAS software, version 9.4. Two-sided tests were applied, with a significance level of *α* = 0.05; a *p* value < 0.05 was considered statistically significant.

## 3 Result

A total of 705 patients who underwent liver biopsies at both baseline and week 72 were included in the analysis. At baseline, 185 patients (26.24%) had Ishak stage 3 fibrosis, 132 (18.72%) had stage 4, 147 (20.85%) had stage 5, and 241 (34.18%) had stage 6. After 72 weeks of treatment, 359 patients (50.90%) achieved fibrosis regression, defined as a ≥ 1-point decrease in Ishak score. When calculated within each baseline stage, 98 (52.97%) of patients with baseline stage 3 achieved regression, 73 (55.30%) with stage 4, 67 (45.58%) with stage 5, and 121 (50.21%) with stage 6. The remaining 346 patients (49.10%) did not achieve regression, including 87 (47.03%) with baseline stage 3, 59 (44.70%) with stage 4, 80 (54.42%) with stage 5, and 120 (49.79%) with stage 6.

### 3.1 Baseline characteristics between fibrosis regression and non-reversal groups

The baseline comparison between the fibrosis regression and non-regression groups is shown in Table 1. Patients with thrombocytopenia (PLT < 100 × 10^9^/L) had a significantly lower likelihood of fibrosis regression compared to those with normal platelet counts (38.10% vs. 53.17%, *p* = 0.004). Similarly, patients with increased spleen thickness (> 40 mm) had a lower regression rate than those with normal spleen thickness (42.75% vs. 52.91%, *p* = 0.032). In addition, the fibrosis regression rate was significantly lower in patients with spleen length > 130 mm compared to those with spleen length ≤ 130 mm (40.00% vs. 52.62%, *p* = 0.022). HBV DNA levels appeared to be lower in the non-regression group compared with the regression group (*p* < 0.001). No statistically significant differences were observed for other baseline variables (*p* > 0.05).

**Table 1:** Baseline characteristics of patients with and without fibrosis regression after entecavir treatment.

Factor (*n*, %)	Category level	Non-regression group (*N* = 346)	Regression group (*N* = 359)	*χ*^2^/Z	*p* value
Age	<45 years	191 (46.59%)	219 (53.41%)	2.44	0.119
	≥45 years	155 (52.54%)	140 (47.46%)		
Sex	Male	233 (48.14%)	251 (51.86%)	0.54	0.461
	Female	113 (51.13%)	108 (48.87%)		
Drinking	Yes	294 (47.80%)	321 (52.20%)	3.12	0.077
	No	52 (57.78%)	38 (42.22%)		
Smoking	Yes	283 (48.29%)	303 (51.71%)	0.85	0.355
	No	63 (52.94%)	56 (47.06%)		
Spleen thickness (mm)	≤40	267 (47.09%)	300 (52.91%)	4.58	0.032
	>40	79 (57.25%)	59 (42.75%)		
Spleen length (mm)	≤130	289 (47.38%)	321 (52.62%)	5.24	0.022
	>130	57 (60.00%)	38 (40.00%)		
BMI	18 ~ 26	265 (47.41%)	294 (52.59%)	3.59	0.166
	<18	9 (47.37%)	10 (52.63%)		
	>26	72 (56.69%)	55 (43.31%)		
WBC (L^−1^, ×10^9^)	3.5 ~ 9.5	308 (48.66%)	325 (51.34%)	3.06	0.216
	>9.5	7 (36.84%)	12 (63.16%)		
	<3.5	31 (58.49%)	22 (41.51%)		
HGB (g·L^−1^)	Male<120; Female<110	346 (49.08%)	359 (50.92%)	3.06	0.216
RBC (L^−1^, ×10^12^)	Male≥4; Female≥3.5	326 (48.58%)	345 (51.42%)	1.36	0.244
	Male<4; Female<3.5	20 (58.82%)	14 (41.18%)		
PLT (L^−1^, ×10^9^)	≥100	281 (46.83%)	319 (53.17%)	8.12	0.004
	<100	65 (61.90%)	40 (38.10%)		
N (L^−1^, ×10^9^)	40 ~ 75	322 (49.46%)	329 (50.54%)	0.57	0.753
	<40	16 (45.71%)	19 (54.29%)		
	>75	8 (42.11%)	11 (57.89%)		
MON (L^−1^, ×10^9^)	≤10	305 (49.59%)	310 (50.41%)	0.51	0.474
	>10	41 (45.56%)	49 (54.44%)		
EOS (L^−1^, ×10^9^)	≤8	340 (49.13%)	352 (50.87%)	0.05	0.831
	>8	6 (46.15%)	7 (53.85%)		
PT (s)	≤15	326 (49.39%)	334 (50.61%)	0.41	0.521
	>15	20 (44.44%)	25 (55.56%)		
APTT (s)	≤35	213 (49.77%)	215 (50.23%)	0.21	0.650
	>35	133 (48.01%)	144 (51.99%)		
TT (s)	≤18	155 (46.97%)	175 (53.03%)	1.10	0.294
	>18	191 (50.93%)	184 (49.07%)		
FIB (g/L)	≥2	255 (49.04%)	265 (50.96%)	0	0.972
	<2	91 (49.19%)	94 (50.81%)		
ALT (U·L^−1^)	≤40	139 (53.88%)	119 (46.12%)	3.75	0.053
	>40	207 (46.31%)	240 (53.69%)		
AST (U·L^−1^)	≤40	161 (50.16%)	160 (49.84%)	0.27	0.601
	>40	185 (48.18%)	199 (51.82%)		
ALP (U·L^−1^)	≤125	299 (47.92%)	325 (52.08%)	2.93	0.087
	>125	47 (58.02%)	34 (41.98%)		
GGT (U·L^−1^)	≤60	239 (47.80%)	261 (52.20%)	1.12	0.289
	>60	107 (52.20%)	98 (47.80%)		
TBIL (μmol·L^−1^)	<17.1	236 (48.76%)	248 (51.24%)	0.61	0.739
	17.1 ~ 34.2	96 (50.79%)	93 (49.21%)		
	>34.2	14 (43.75%)	18 (56.25%)		
GLO (g·L^−1^)	20 ~ 40	329 (49.03%)	342 (50.97%)		0.952
	<20	2 (40.00%)	3 (60.00%)		
	>40	15 (51.72%)	14 (48.28%)		
ALB (g·L^−1^)	≥40	246 (48.71%)	259 (51.29%)	0.09	0.758
	<40	100 (50.00%)	100 (50.00%)		
CR (μmol·L^−1^)	Male≤115; Female≤97	346 (49.22%)	357 (50.78%)		0.500
	Male>115; Female>97	0 (0%)	2 (100%)		
BUN (mmol·L^−1^)	3.2 ~ 7.1	298 (49.50%)	304 (50.50%)	3.17	0.205
	<3.2	34 (53.13%)	30 (46.88%)		
	>7.1	14 (35.90%)	25 (64.10%)		
AFP (ng·mL^−1^)	≤25	301 (50.17%)	299 (49.83%)	1.91	0.167
	>25	45 (42.86%)	60 (57.14%)		
HBeAg	Positive	283 (48.29%)	303 (51.71%)	0.85	0.355
	Negative	63 (52.94%)	56 (47.06%)		
HBeAb	Positive	283 (48.29%)	303 (51.71%)	0.85	0.355
	Negative	63 (52.94%)	56 (47.06%)		
Inflammation grade	3–6	154 (50.83%)	149 (49.17%)	0.69	0.710
	7–10	167 (47.58%)	184 (52.42%)		
	11–14	25 (49.02%)	26 (50.98%)		
Fibrosis	Ishak 3	87 (25.14%)	98 (27.30%)	3.05	0.383
	Ishak 4	59 (17.05%)	73 (20.33%)		
	Ishak 5	80 (23.12%)	67 (18.66%)		
	Ishak 6	120 (34.68%)	121 (33.70%)		
HBV DNA (Log_10_IU·mol^−1^)		5.76 (4.64, 7.23)	6.46 (4.98, 7.73)	−3.40	<0.001

### 3.2 Effect of baseline fibrosis stage on fibrosis regression

**Figure 1:**
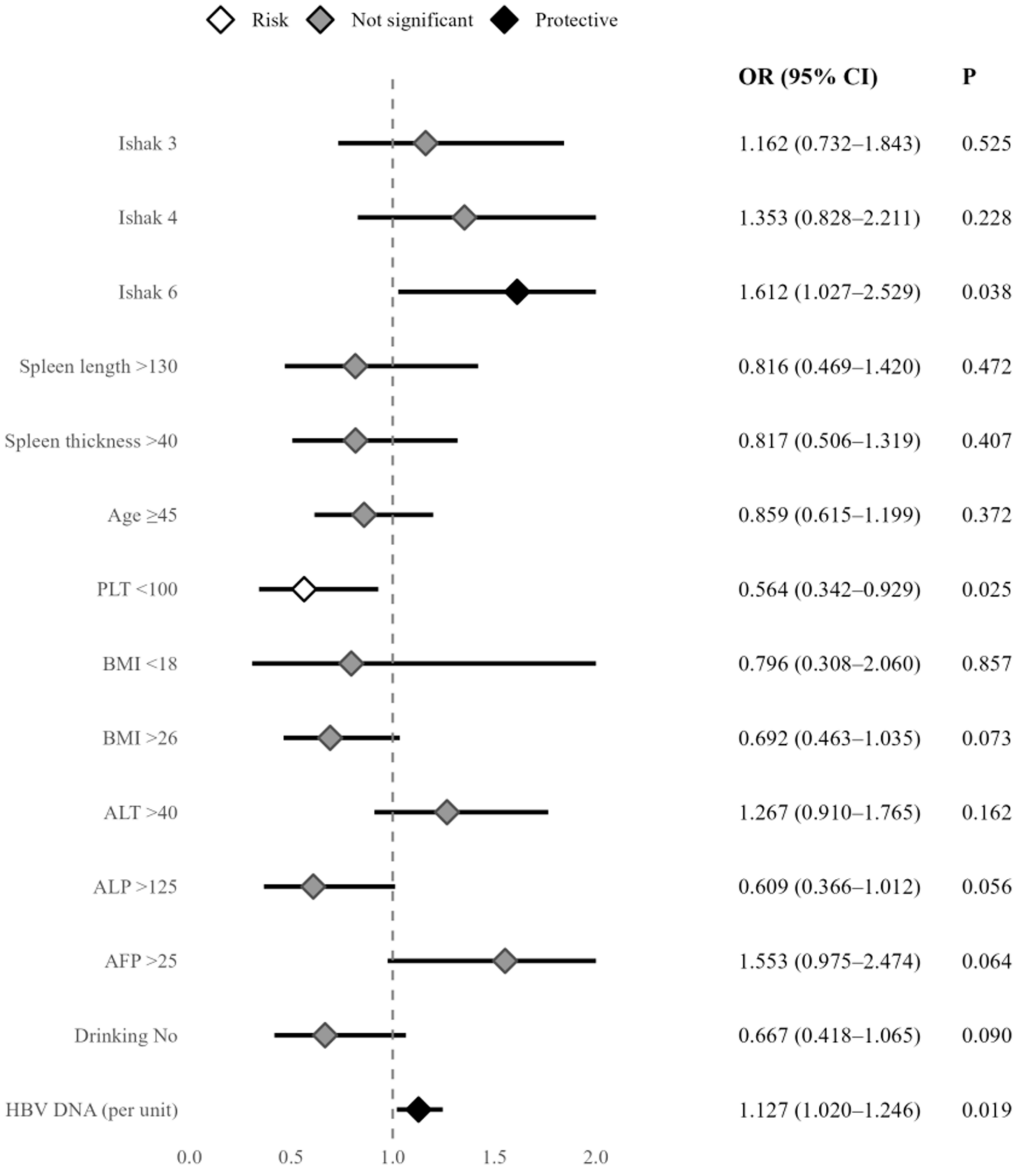
Multiple logistic regression analysis of reversal factors of liver fibrosis. Risk indicates variables with OR < 1 and *p* < 0.05, suggesting a decreased likelihood of fibrosis regression. Protective indicates variables with OR > 1 and *p* < 0.05, suggesting an increased likelihood of fibrosis regression. Not significant refers to variables with *p* ≥ 0.05.

To assess the impact of baseline fibrosis stage on fibrosis regression, multivariable logistic regression analysis was performed. Based on baseline comparisons (*p* < 0.2, Table 1), 11 covariates were included: age, alcohol use, spleen length, spleen thickness, PLT, BMI, ALT, ALP, AFP, HBV DNA level, and baseline Ishak fibrosis stage. Fibrosis regression (yes/no) was defined as the dependent variable, with treatment regimen (entecavir alone vs. entecavir plus Biejia-Ruangan) set as the stratification factor. After adjustment for the above covariates, patients with baseline Ishak stage 6 were more likely to achieve fibrosis regression compared to those with stage 5(*OR*:1.612, *95%CI*:1.027 ~ 2.529, *p* = 0.038). (See Table 2 and Figure 1). To further contextualize the clinical relevance of this finding, the absolute risk difference (ARD) in histological reversal rates between patients with Ishak stage 6 (50.21%) and those with stage 5 (45.58%) was 4.63%. This corresponds to an estimated number needed to treat (NNT) of approximately 22, suggesting that, in this cohort, for every 22 patients with Ishak stage 6 receiving long-term antiviral therapy, one additional patient may achieve measurable histological downgrading compared with those starting at stage 5.

**Table 2:** Logistic regression analysis results of factors related to liver fibrosis regression.

Factor	Category level	*b*	SE	OR (95% CI)	*χ* ^2^	*p* value
Fibrosis	Ishak 5	Reference		1		
	Ishak 3	0.150	0.236	1.162 (0.732, 1.843)	0.404	0.525
	Ishak 4	0.302	0.251	1.353 (0.828, 2.211)	1.452	0.228
	Ishak 6	0.477	0.230	1.612 (1.027, 2.529)	4.313	0.038
Spleen length	≤130	Reference		1		
	>130	−0.204	0.283	0.816 (0.469, 1.420)	0.518	0.472
Spleen thickness	≤40	Reference		1		
	>40	−0.203	0.245	0.817 (0.506, 1.319)	0.687	0.407
Age	<45	Reference		1		
	≥45	−0.152	0.170	0.859 (0.615, 1.199)	0.797	0.372
PLT (L^−1^, ×10^9^)	≥100	Reference		1		
	<100	−0.573	0.255	0.564 (0.342, 0.929)	5.047	0.025
BMI	18 ~ 26	Reference		1		
	<18	−0.228	0.485	0.796 (0.308, 2.060)	0.221	0.857
	>26	−0.368	0.205	0.692 (0.463, 1.035)	3.205	0.073
ALT (U·L^−1^)	≤40	Reference		1		
	>40	0.237	0.169	1.267 (0.910, 1.765)	1.960	0.162
ALP (U·L^−1^)	≤125	Reference		1		
	>125	−0.497	0.260	0.609 (0.366, 1.012)	3.660	0.056
AFP (ng·mL^−1^)	≤25	Reference		1		
	>25	0.440	0.238	1.553 (0.975, 2.474)	3.429	0.064
Drinking	Yes			1		
	No	−0.405	0.239	0.667 (0.418, 1.065)	2.876	0.090
HBVDNA (Log_10_IU·mol)		0.120	0.051	1.127 (1.020, 1.246)	5.482	0.019

### 3.3 Non-linear trajectory of fibrosis regression across Ishak stages

**Figure 2:**
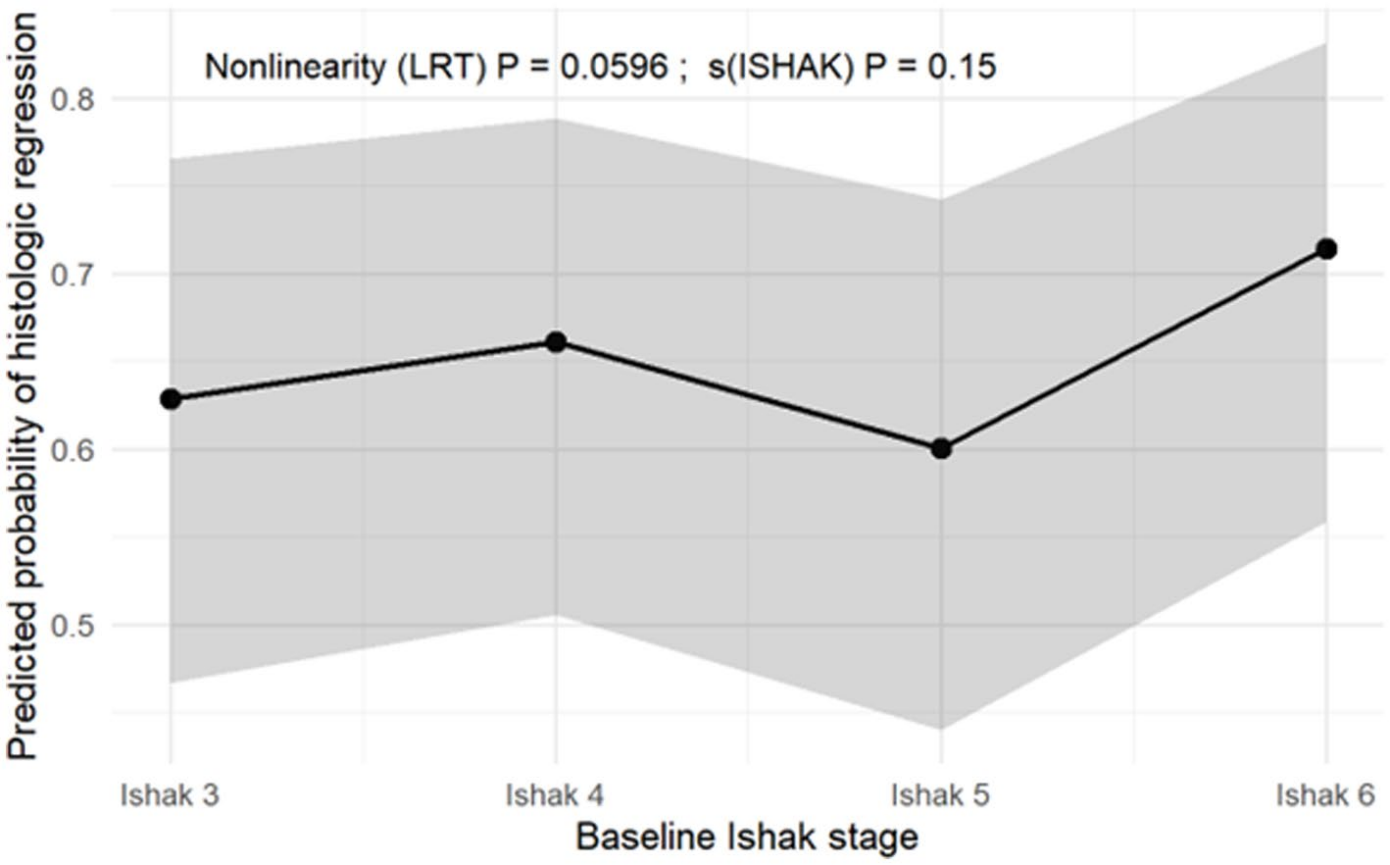
Non-linear trend of fibrosis regression across baseline Ishak stages (GAM adjusted). The solid line represents the estimated probability of regression based on the generalized additive model (GAM). The shaded area represents the 95% confidence interval (CI).

In the generalized additive model (GAM) adjusted for the same covariates as the logistic regression, the probability of histologic regression exhibited a non-linear but statistically non-significant trend across Ishak stages [LRT *p* = 0.0596; s(ISHAK) *p* = 0.15] (Figure 2). The predicted probability of regression slightly increased from Ishak stages 3 to 4, decreased at stage 5, and rose again at stage 6. These findings suggest that fibrosis regression may not decline strictly linearly with increasing baseline severity. Instead, patients with baseline stage 6 appeared to have a comparable or slightly higher adjusted probability of regression compared with those at stage 5. These results should be interpreted as exploratory.

### 3.4 Sensitivity analysis

To further validate the robustness of the primary findings, a sensitivity analysis was conducted in patients with baseline Ishak stage 5 or 6 (*n* = 388), selected from the overall cohort of 705 patients. A sensitivity analysis was conducted using fibrosis regression (yes/no) at week 72 as the binary dependent variable. Baseline characteristics of patients with and without fibrosis regression in the Ishak stage 5 and 6 subgroup are shown in Table 3.

**Table 3:** Univariate analysis of factors associated with fibrosis regression in patients with baseline Ishak stage 5–6.

Factor (*n*, %)	Category level	Non-regression group (*N* = 200)	Regression group (*N* = 188)	*χ*^2^/Z	*p* value
Age	<45 years	80 (43.72%)	103 (56.28%)	8.50	0.004
	≥45 years	120 (58.54%)	85 (41.46%)		
Sex	Male	139 (48.94%)	145 (51.06%)	2.87	0.090
	Female	61 (58.65%)	43 (41.35%)		
Drinking	Yes	162 (49.24%)	167 (50.76%)	4.61	0.032
	No	38 (64.41%)	21 (35.59%)		
Smoking	Yes	156 (49.84%)	157 (50.16%)	1.89	0.170
	No	44 (58.67%)	31 (41.33%)		
Spleen thickness	≤40	135 (49.09%)	140 (50.91%)	2.28	0.131
	>40	65 (57.52%)	48 (42.48%)		
Spleen length	≤130	151 (48.87%)	158 (51.13%)	4.36	0.037
	>130	49 (62.03%)	30 (37.97%)		
BMI	18 ~ 26	154 (50.66%)	150 (49.34%)		0.786
	<18	4 (57.14%)	3 (42.86%)		
	>26	42 (54.55%)	35 (45.45%)		
WBC (L^−1^, ×10^9^)	3.5 ~ 9.5	170 (50.60%)	166 (49.40%)	1.74	0.418
	>9.5	5 (45.45%)	6 (54.55%)		
	<3.5	25 (60.98%)	16 (39.02%)		
HGB (g·L^−1^)	Male<120; Female<110	200 (51.55%)	188 (48.45%)	1.74	0.418
RBC (L^−1^, ×10^12^)	Male≥4; Female≥3.5	184 (50.83%)	178 (49.17%)	1.11	0.291
	Male<4; Female<3.5	16 (61.54%)	10 (38.46%)		
PLT (L^−1^, ×10^9^)	≥100	141 (48.45%)	150 (51.55%)	4.46	0.035
	<100	59 (60.82%)	38 (39.18%)		
N (L^−1^, ×10^9^)	40 ~ 75	186 (51.81%)	173 (48.19%)		0.549
	<40	10 (43.48%)	13 (56.52%)		
	>75	4 (66.67%)	2 (33.33%)		
MON (L^−1^, ×10^9^)	≤10	175 (51.62%)	164 (48.38%)	0.01	0.937
	>10	25 (51.02%)	24 (48.98%)		
EOS (L^−1^, ×10^9^)	≤8	195 (51.32%)	185 (48.68%)		0.725
	>8	5 (62.50%)	3 (37.50%)		
PT (s)	≤15	183 (51.55%)	172 (48.45%)	0	0.997
	>15	17 (51.52%)	16 (48.48%)		
APTT (s)	≤35	113 (50.00%)	113 (50.00%)	0.52	0.472
	>35	87 (53.70%)	75 (46.30%)		
TT (s)	≤18	84 (52.83%)	75 (47.17%)	0.18	0.673
	>18	116 (50.66%)	113 (49.34%)		
FIB (g·L^−1^)	≥2	142 (51.82%)	132 (48.18%)	0.03	0.865
	<2	58 (50.88%)	56 (49.12%)		
ALT (U·L^−1^)	≤40	79 (54.48%)	66 (45.52%)	0.8	0.371
	>40	121 (49.79%)	122 (50.21%)		
AST (U·L^−1^)	≤40	88 (52.69%)	79 (47.31%)	0.15	0.694
	>40	112 (50.68%)	109 (49.32%)		
ALP (U·L^−1^)	≤125	164 (49.85%)	165 (50.15%)	2.5	0.114
	>125	36 (61.02%)	23 (38.98%)		
GGT (U·L^−1^)	≤60	122 (50.00%)	122 (50.00%)	0.63	0.428
	>60	78 (54.17%)	66 (45.83%)		
TBIL (μmol·L^−1^)	<17.1	115 (48.94%)	120 (51.06%)	1.98	0.37
	17.1 ~ 34.2	73 (56.59%)	56 (43.41%)		
	>34.2	12 (50.00%)	12 (50.00%)		
GLO (g·L^−1^)	20 ~ 40	188 (51.65%)	176 (48.35%)		1
	<20	2 (50.00%)	2 (50.00%)		
	>40	10 (50.00%)	10 (50.00%)		
ALB (g·L^−1^)	≥40	128 (50.00%)	128 (50.00%)	0.72	0.396
	<40	72 (54.55%)	60 (45.45%)		
CR (μmol·L^−1^)	Male≤115; Female≤97	200 (51.81%)	186 (48.19%)		0.2341
	Male>115; Female>97	0 (0%)	2 (100%)		
BUN (mmol·L^−1^)	3.2 ~ 7.1	173 (52.11%)	159 (47.89%)	2.14	0.343
	<3.2	17 (56.67%)	13 (43.33%)		
	>7.1	10 (38.46%)	16 (61.54%)		
AFP (ng·mL^−1^)	≤25	161 (52.27%)	147 (47.73%)	0.32	0.574
	>25	39 (48.75%)	41 (51.25%)		
HBeAg	Positive	156 (49.84%)	157 (50.16%)	1.89	0.170
	Negative	44 (58.67%)	31 (41.33%)		
HBeAb	Positive	156 (49.84%)	157 (50.16%)	1.89	0.170
	Negative	44 (58.67%)	31 (41.33%)		
Inflammation grade	1	62 (55.36%)	50 (44.64%)	1.04	0.595
	2	120 (50.42%)	118 (49.58%)		
	3	18 (47.37%)	20 (52.63%)		
Fibrosis	Ishak 5	80 (54.42%)	67 (45.58%)	0.78	0.3761
	Ishak 6	120 (49.79%)	121 (50.21%)		
HBVDNA (Log_10_IU·mol)		5.42 (4.63,6.76)	6.01 (4.80,7.23)	2.46	0.014

Twelve variables with *p* < 0.20 in the univariate analysis were selected from Table 3, including fibrosis stage, spleen thickness (spleenh), spleen length (spleenl), age, sex, PLT, ALP, Drinking, Smoking, HBeAg, HBeAb, and HBV DNA level. These variables were entered into a multivariable logistic regression model.

After adjusting for these covariates, patients with baseline Ishak stage 6 had a significantly higher likelihood of achieving fibrosis regression compared to those with stage 5 (*OR*: 1.835, *95%CI*: 1.148 ~ 2.931, *p* = 0.011). (See Table 4).

**Table 4:** Multivariable logistic regression analysis of factors associated with fibrosis regression in patients with baseline Ishak stage 5–6.

Factor	Category level	*b*	SE	OR (95% CI)	*χ* ^2^	*p* value
Fibrosis	Ishak 5	Reference		1		
	Ishak 6	0.607	0.239	1.835 (1.148, 2.931)	6.437	0.011
Spleen thickness	≤40	Reference		1		
	>40	−0.207	0.294	0.813 (0.457, 1.446)	0.497	0.481
Spleen length	≤130	Reference		1		
	>130	−0.307	0.332	0.736 (0.384, 1.411)	0.854	0.355
Age	<45	Reference		1		
	≥45	−0.595	0.229	0.552 (0.352, 0.864)	6.744	0.009
Sex	Male	Reference		1		
	Female	−0.413	0.263	0.661 (0.395, 1.107)	2.476	0.116
PLT (L^−1^, ×10^9^)	≥100	Reference		1		
	<100	−0.423	0.275	0.655 (0.382, 1.122)	2.375	0.123
ALP (U·L^−1^)	≤125	Reference		1		
	>125	−0.002	0.003	0.998 (0.992, 1.004)	0.440	0.507
Drinking	Yes	Reference		1		
	No	−0.617	0.355	0.540 (0.269, 1.083)	3.015	0.083
Smoking	Yes	Reference		1		
	No	−0.271	0.331	0.763 (0.399, 1.458)	0.670	0.413
HBeAg	Positive	Reference		1		
	Negative	−0.045	0.305	0.956 (0.526, 1.736)	0.022	0.883
HBeAb	Positive	Reference				
	Negative	−0.134	0.308	0.875 (0.478, 1.600)	0.189	0.664
HBVDNA (Log_10_IU·mol^−1^)		0.126	0.080	1.135 (0.970, 1.328)	2.482	0.115

### 3.5 Subgroup analyses stratified by treatment

To evaluate whether concomitant use of Biejia-Ruangan (BR) influenced the association between baseline Ishak stage and fibrosis regression, subgroup analyses were performed separately in patients receiving ETV monotherapy and those receiving ETV + BR. As shown in Supplementary Table S1, the direction of association remained consistent across both treatment groups: patients with baseline Ishak stage 6 demonstrated a higher—though not statistically significant—likelihood of regression compared with those at stage 5 in both the ETV group (*OR* = 1.917*, 95%CI:* 0.941 ~ 3.903*, p* = 0.073) and the ETV + BR group (OR = 1.452, 95%CI: 0.785 ~ 2.687*, p* = 0.235). These findings indicate that BR did not appear to substantially modify the stage-related differences in regression probability.

## 4 Discussion

The primary goal of chronic hepatitis B (CHB) management is to achieve sustained viral suppression, thereby controlling liver fibrosis and preventing progression to clinical complications such as hepatic decompensation and hepatocellular carcinoma (HCC) (11, 12). A durable virological response has been shown to delay, and in some cases even reverse, hepatic fibrosis (13, 14). However, data on the potential for fibrosis regression across different stages of cirrhosis—particularly in advanced stages—remain limited. In this context, our analysis of paired liver biopsies from CHB patients treated with entecavir found that individuals with baseline Ishak stage 6 fibrosis appeared to have a somewhat higher probability of histological regression than those with stage 5. This observation is not entirely consistent with the conventional assumption that less advanced fibrosis is invariably more reversible. Instead, our data suggest that regression in advanced disease may follow a stage-dependent course, in which varying degrees of architectural distortion do not correspond to a simple linear decline in reversibility.

Given prior evidence suggesting that Biejia–Ruangan (BR) may exert anti-fibrotic effects, we conducted stratified analyses according to treatment regimen. As shown in Supplementary Tables S2, S4, the trend toward a higher likelihood of regression in patients with baseline Ishak stage 6 compared with stage 5 was consistent in both the ETV group and the ETV + BR group, and concomitant BR use did not appear to substantially modify this stage-related difference. Given the relatively small sample sizes within these subgroups, the precision of the effect estimates may be limited. Therefore, the observed disparity between Ishak stages is more likely attributable to intrinsic histological reversibility rather than treatment-specific effects.

One plausible explanation for this finding is that regression from Ishak stage 6 to stage 5 may involve relatively modest architectural modifications—such as partial thinning or resorption of fibrous septa and remodeling of small regenerative nodules—which have been described in morphologic studies of cirrhosis undergoing partial reversal (15–19). By contrast, regression to pre-cirrhotic stages requires extensive remodeling, including disruption of bridging septa and restoration of lobular architecture—a process that is biologically more complex and far less frequently achieved (20, 21). These findings are consistent with the concept that Ishak stage 5 encompasses substantial histological heterogeneity, which may limit the likelihood of further regression, whereas stage 6, despite indicating more advanced disease, requires a lower threshold of structural change to achieve measurable downgrading within categorical scoring systems. In addition, Ishak stage 5 may represent a more structurally stable transitional stage, in which persistent bridging fibrosis may limit further regression. An alternative explanation for the higher apparent regression rate observed in patients with Ishak stage 6 is regression to the mean, whereby individuals with more extreme baseline values tend to show greater apparent improvement on follow-up. Although ‘regression to the mean’ cannot be entirely ruled out as a statistical factor, the support from existing pathological evidence leads us to consider this a real biological phenomenon.

Our findings also highlight the dichotomy within advanced fibrosis. Although patients with stage 6 were more likely to regress to stage 5, markers of decompensation such as splenomegaly and thrombocytopenia were associated with reduced likelihood of improvement, consistent with previous reports linking portal hypertension and hypersplenism to limited histological recovery (22). Moreover, in the overall cohort, higher baseline HBV DNA levels were significantly associated with histological regression (*p* < 0.001), whereas this association was not observed when the analysis was restricted to patients with Ishak stages 5–6 (*p* = 0.115). This suggests that the determinants of fibrosis regression may differ by disease stage: in earlier stages, virological activity may exert a stronger influence, while in advanced fibrosis, structural remodeling appears to play a more dominant role. Nevertheless, these findings should be interpreted with caution and require validation in independent prospective cohorts.

Finally, current evidence indicates that fibrosis progression and regression do not follow a simple linear course, and this non-linearity is not adequately captured by categorical systems such as the Ishak score (15, 23). Whereas progression is primarily driven by inflammation-induced collagen deposition and architectural distortion, regression depends on suppression of fibrogenic pathways, activation of matrix degradation, and restoration of parenchymal and vascular architecture (21, 24). These processes occur to variable degrees across histological stages, explaining the stage-dependent reversibility observed in the present study. Even among patients with advanced fibrosis, long-term antiviral therapy may still promote limited histological improvement, though the extent of regression is likely constrained by pathological heterogeneity, collagen cross-linking, and the presence of clinical decompensation.

A substantial body of high-quality clinical evidence has established that entecavir (ETV), a potent nucleoside analogue, can profoundly suppress hepatitis B virus (HBV) DNA replication, resulting in a marked reduction in viral load. This virological suppression not only attenuates hepatic inflammation but also improves the hepatic microenvironment and facilitates histological regression of fibrotic architecture (25, 26). Although histological reversal of liver fibrosis or cirrhosis following antiviral therapy has been reported in patients with chronic hepatitis B (CHB), most of these studies have included only a small number of individuals with advanced-stage fibrosis. Given that late-stage fibrosis is typically accompanied by substantial structural distortion and impaired intrahepatic hemodynamics, its reversibility remains a subject of ongoing debate. Clarifying whether patients with advanced fibrosis can similarly benefit from antiviral therapy is of considerable clinical importance and may have implications for therapeutic decision-making.

Accumulating evidence now indicates that fibrosis regression is influenced by the underlying fibrosis stage. Sun et al. (27) demonstrated that semi-quantitative systems such as the Ishak score are insufficient to reflect the subtle and continuous histologic changes that occur during fibrosis regression. Lee et al. (28) and Khan et al. (29) further described fibrosis regression as a multi-step process characterized by septal thinning, fragmentation, and vascular remodeling that vary in degree among different stages of fibrosis. In addition, Chen et al. (30) also observed a biphasic regression pattern over the treatment course, indicating that fibrosis regression may proceed in a non-linear manner over time, consistent with the stage-related heterogeneity observed in our study. However, most previous studies have not systematically stratified treatment outcomes according to different stages of hepatic fibrosis.

The strengths of this study lie in its multicenter, randomized controlled trial (RCT) design, which enabled the inclusion of a more representative patient population across diverse regions and healthcare settings, thereby enhancing the generalizability of the findings. Rigorous randomization and allocation concealment effectively minimized potential confounding factors, particularly by reducing the risk of bias from physicians potentially providing additional interventions or care to patients with Ishak stage 6 fibrosis. Furthermore, the relatively large sample size ensured sufficient statistical power for stratified analyses and multivariate modeling, contributing to the robustness and reliability of both the statistical and clinical interpretations.

In interpreting these findings, it should be acknowledged that the Ishak scoring system is a semi-quantitative scale and may not fully capture the continuous and spatially heterogeneous nature of fibrosis remodeling. Although quantitative morphometric approaches, such as qFibrosis and collagen proportionate area analysis, provide more granular assessment of collagen architecture, such methods were not available in this cohort. Consequently, subtle microstructural changes may not have been fully reflected in categorical stage downgrading.

In this study, the difference in histological reversal rates between patients with Ishak stage 6 and those with stage 5 fibrosis was statistically significant (*OR*:1.612, *95%CI*:1.027 ~ 2.529, *p* = 0.038). However, the relatively wide confidence interval indicates that the magnitude of this association should be interpreted with caution. In absolute terms, the unadjusted proportion of histological regression was modestly higher in patients with Ishak stage 6 than in those with stage 5 (50.2% vs. 45.6%), corresponding to an absolute difference of approximately 4%–5%. From a clinical perspective, these findings indicate that patients with advanced-stage disease may still derive histological benefit from sustained antiviral therapy. Clinicians can use these data to reassure patients with established cirrhosis that their liver architecture is not strictly irreversible, thereby reinforcing the critical importance of strict, long-term adherence to antiviral and anti-fibrotic therapies.

Several limitations of this study should be acknowledged. First, the follow-up duration was limited to 72 weeks. Although this time frame is sufficient to detect short- to mid-term histological changes, longer follow-up may reveal different patterns of fibrosis regression and provide more robust prognostic information. Second, all participants were recruited from multiple centers in China, which may limit the generalizability of our findings to other populations and healthcare settings. Third, fibrosis was assessed using the Ishak staging system, which is a semi-quantitative categorical scale and may not fully capture the continuous and spatially heterogeneous nature of fibrosis remodeling. More quantitative morphometric approaches were not available in this cohort. In addition, this study did not integrate complementary imaging-based assessments (e.g., elastography), which may provide additional mechanistic insights into stage-dependent differences. Fourth, this study did not systematically link histological regression to hard clinical outcomes, such as hepatic decompensation, portal hypertension–related complications, or hepatocellular carcinoma. Therefore, the long-term prognostic significance of biopsy-based downgrading in advanced fibrosis remains to be fully clarified. Fifth, despite strict eligibility criteria and adjustment for major clinical and histological covariates, residual confounding cannot be entirely excluded due to unmeasured or incompletely captured variables, including metabolic comorbidities. Additionally, this study focused solely on the histological reversal of fibrosis following long-term antiviral therapy in patients with hepatitis B–related cirrhosis. The relationship between histological improvement and long-term clinical outcomes remains unclear and warrants further investigation through large-scale, prospective studies with extended follow-up.

## 5 Conclusion

In conclusion, this study indicates that in patients with chronic hepatitis B receiving entecavir therapy, fibrosis regression follows a non-linear, stage-related pattern. Patients with baseline Ishak stage 6 fibrosis showed a higher rate of histological regression compared with those at stage 5, supporting the concept that reversibility in advanced fibrosis is influenced by structural stage rather than baseline severity alone. Further studies with extended follow-up durations and incorporation of quantitative histological assessments are warranted to validate these findings and better inform fibrosis staging and therapeutic decision-making in clinical practice.

## Data Availability

The original contributions presented in the study are included in the article/Supplementary material, further inquiries can be directed to the corresponding authors.
