# Supplementary material for: Ishak stage 6 fibrosis is more likely to regress than stage 5 in CHB patients undergoing entecavir therapy
Source: Front Med (Lausanne). 2026 Mar 9;13:1765594. doi: 10.3389/fmed.2026.1765594 (PMC13006690; doi:10.3389/fmed.2026.1765594)
Supplement: Supplementary file 1 [file Table_1.docx]

**Supplementary Table 1 Baseline characteristics between fibrosis reversal and non-reversal groups in patients treated with ETV+Placebo**

| Factor(n, %) | Category Level | Non-regression group(N=106) | Regression group(N=78) | | X^2^/Z | *P* value |
| --- | --- | --- | --- | --- | --- | --- |
| AGE | <45 years | 36(47.37%) | 40(52.63%) | 5.56 | | 0.018 |
|  | ≥45 years | 70(64.81%) | 38(35.19%) |  | |  |
| SEX | Male | 73(54.89%) | 60(45.11%) | 1.46 | | 0.228 |
|  | Female | 33(64.71%) | 18(35.29%) |  | |  |
| Drinking | Yes | 85(55.19%) | 69(44.81%) | 2.25 | | 0.133 |
|  | No | 21(70%) | 9(30%) |  | |  |
| Smoking | Yes | 80(55.56%) | 64(44.44%) | 1.14 | | 0.285 |
|  | No | 26(65%) | 14(35%) |  | |  |
| Spleen thickness(mm) | ≤40 | 71(55.04%) | 58(44.96%) | 1.17 | | 0.280 |
|  | >40 | 35(63.64%) | 20(36.36%) |  | |  |
| Spleen length(mm) | ≤130 | 78(54.17%) | 66(45.83%) | 3.21 | | 0.070 |
|  | >130 | 28(70%) | 12(30%) |  | |  |
| BMI | 18～26 | 76(55.88%) | 60(44.12%) |  | | 0.819 |
|  | ＜18 | 3(60%) | 2(40%) |  | |  |
|  | ＞26 | 27(62.79%) | 16(37.21%) |  | |  |
| WBC (L^−1^, ×10⁹) | 3.5～9.5 | 90(57.32%) | 67(42.68%) |  | | 0.345 |
|  | ＞9.5 | 3(37.5%) | 5(62.5%) |  | |  |
|  | ＜3.5 | 13(68.42%) | 6(31.58%) |  | |  |
| HGB(g·L^−1^) | Male＜120; Female＜110 | 102(58.96%) | 71(41.04%) |  | | 0.208 |
|  |  | 4(36.36%) | 7(63.64%) |  | |  |
| RBC (L^−1^, ×10¹²) | Male≥4; Female≥3.5 | 100(57.8%) | 73(42.2%) |  | | 1 |
|  | Male＜4; Female＜3.5 | 6(54.55%) | 5(45.45%) |  | |  |
| PLT (L^−1^, ×10⁹) | ≥100 | 71(52.21%) | 65(47.79%) | 6.23 | | 0.013 |
|  | ＜100 | 35(72.92%) | 13(27.08%) |  | |  |
| N(L^−1^, ×10⁹) | 40～75 | 99(58.93%) | 69(41.07%) |  | | 0.443 |
|  | ＜40 | 6(42.86%) | 8(57.14%) |  | |  |
|  | ＞75 | 1(50%) | 1(50%) |  | |  |
| MON (L^−1^, ×10⁹) | ≤10 | 91(58.71%) | 64(41.29%) | 0.49 | | 0.485 |
|  | ＞10 | 15(51.72%) | 14(48.28%) |  | |  |
| EOS (L^−1^, ×10⁹) | ≤8 | 104(57.14%) | 78(42.86%) |  | | 0.509 |
|  | ＞8 | 2(100%) | 0(0%) |  | |  |
| PT(s) | ≤15 | 101(58.05%) | 73(41.95%) |  | | 0.745 |
|  | ＞15 | 5(50%) | 5(50%) |  | |  |
| APTT(s) | ≤35 | 60(55.56%) | 48(44.44%) | 0.45 | | 0.502 |
|  | ＞35 | 46(60.53%) | 30(39.47%) |  | |  |
| TT(s) | ≤18 | 51(62.2%) | 31(37.8%) | 1.27 | | 0.259 |
|  | ＞18 | 55(53.92%) | 47(46.08%) |  | |  |
| FIB(g/L) | ≥2 | 72(55.38%) | 58(44.62%) | 0.9 | | 0.344 |
|  | ＜2 | 34(62.96%) | 20(37.04%) |  | |  |
| ALT(U·L^−1^) | ≤40 | 38(55.88%) | 30(44.12%) | 0.13 | | 0.717 |
|  | ＞40 | 68(58.62%) | 48(41.38%) |  | |  |
| AST(U·L^−1^) | ≤40 | 46(54.76%) | 38(45.24%) | 0.51 | | 0.474 |
|  | ＞40 | 60(60%) | 40(40%) |  | |  |
| ALP(U·L^−1^) | ≤125 | 87(55.77%) | 69(44.23%) | 1.42 | | 0.233 |
|  | ＞125 | 19(67.86%) | 9(32.14%) |  | |  |
| GGT(U·L^−1^) | ≤60 | 59(52.21%) | 54(47.79%) | 3.49 | | 0.062 |
|  | ＞60 | 47(66.2%) | 24(33.8%) |  | | . |
| TBIL(μmol·L^−1^) | ＜17.1 | 59(55.14%) | 48(44.86%) |  | | 0.559 |
|  | 17.1～34.2 | 42(62.69%) | 25(37.31%) |  | |  |
|  | ＞34.2 | 5(50%) | 5(50%) |  | |  |
| GLO(g·L^−1^) | 20～40 | 101(59.41%) | 69(40.59%) |  | | 0.170 |
|  | ＜20 | 1(50%) | 1(50%) |  | |  |
|  | ＞40 | 4(33.33%) | 8(66.67%) |  | |  |
| ALB(g·L^−1^) | ≥40 | 65(54.17%) | 55(45.83%) | 1.67 | | 0.196 |
|  | ＜40 | 41(64.06%) | 23(35.94%) |  | |  |
| CR(μmol·L^−1^) | Male≤115; Female≤97 | 65(54.17%) | 55(45.83%) | 1.67 | | 0.196 |
|  | Male＞115; Female＞97 | 41(64.06%) | 23(35.94%) |  | |  |
| BUN(mmol·L^−1^) | 3.2～7.1 | 92(59.74%) | 62(40.26%) | 2.87 | | 0.238 |
|  | ＜3.2 | 7(58.33%) | 5(41.67%) |  | |  |
|  | ＞7.1 | 7(38.89%) | 11(61.11%) |  | |  |
| AFP(ng·mL^−1^) | ≤25 | 84(58.33%) | 60(41.67%) | 0.14 | | 0.706 |
|  | ＞25 | 22(55%) | 18(45%) |  | |  |
| HBeAg | positive | 80(55.56%) | 64(44.44%) | 1.14 | | 0.285 |
|  | negative | 26(65%) | 14(35%) |  | |  |
| HBeAb | positive | 80(55.56%) | 64(44.44%) | 1.14 | | 0.285 |
|  | negative | 26(65%) | 14(35%) |  | |  |
| inflammation grade | 3-6 | 28(54.9%) | 23(45.1%) | 0.39 | | 0.822 |
|  | 7-10 | 66(59.46%) | 45(40.54%) |  | |  |
|  | 11-14 | 12(54.55%) | 10(45.45%) |  | |  |
| Fibrosis | Ishak 5 | 42(60%) | 28(40%) | 0.26 | | 0.607 |
|  | Ishak 6 | 64(56.14%) | 50(43.86%) |  | |  |
| HBV DNA(Log_10_IU·mol^−1^) |  | 5.55(4.70, 6.65) | 6.00(4.87,7.30) | 1.14 | | 0.071 |

**Supplementary Table 2 Multivariate Logistic Regression Analysis of Factors Associated with Fibrosis Reversal in CHB Patients Treated with Entecavir Alone**

| Factor | Category Level | b | SE | OR(95% CI) | X^2^ | P value |
| --- | --- | --- | --- | --- | --- | --- |
| Fibrosis | Ishak 5 | Reference | | 1 |  |  |
|  | Ishak 6 | 0.651 | 0.363 | 1.917(0.941,3.903) | 3.216 | 0.073 |
| Spleen length | ≤130 | Reference |  | 1 |  |  |
|  | >130 | -0.204 | 0.283 | 0.628(0.228,1.730) | 0.518 | 0.472 |
| Spleen thickness | ≤40 | Reference |  | 1 |  |  |
|  | >40 | -0.203 | 0.245 | 0.833(0.333,2.083) | 0.687 | 0.407 |
| AGE | <45 | Reference |  | 1 |  |  |
|  | ≥45 | -1.026 | 0.354 | 0.358(0.179,0.717) | 8.417 | 0.004 |
| PLT(L^−1^, ×10⁹) | ≥100 | Reference |  | 1 |  |  |
|  | ＜100 | -0.917 | 0.425 | 0.400(0.174,0.920) | 4.649 | 0.031 |
| GGT(U·L^−1^) | ≤60 | Reference |  | 1 |  |  |
|  | ＞60 | -0.815 | 0.363 | 0.443(0.217,0.901) | 5.045 | 0.025 |
| GLO(g·L^−1^) | 20～40 | Reference |  | 1 |  |  |
|  | ＜20 | 0.960 | 1.544 | 2.611(0.127,53.869) | 0.386 | 0.534 |
|  | ＞40 | 1.539 | 0.722 | 4.658(1.131,19.193) | 4.537 | 0.033 |
| ALB(U·L^−1^) | ≥40 | Reference |  | 1 |  |  |
|  | ＜40 | -0.368 | 0.376 | 0.692(0.331,1.445) | 0.961 | 0.327 |
| Dirnking | YES | Reference |  | 1 |  |  |
|  | NO | -0.577 | 0.490 | 0.562(0.215,1.469) | 1.381 | 0.240 |
| HBVDNA(Log_10_IU·mol) |  | 0.175 | 0.115 | 1.191(0.950,1.493) | 2.291 | 0.130 |

**Supplementary Table 3 Baseline characteristics between fibrosis reversal and non-reversal groups in patients treated with Entecavir Plus Biejia-Ruangan**

| Factor(n, %) | Category Level | Non-regression group(N=94) | Regression group(N=110) | | X^2^/Z | *P* value |
| --- | --- | --- | --- | --- | --- | --- |
| AGE | <45 years | 44(41.12%) | 63(58.88%) | 2.23 | | 0.136 |
|  | ≥45 years | 50(51.55%) | 47(48.45%) |  | |  |
| SEX | Male | 66(43.71%) | 85(56.29%) | 1.31 | | 0.252 |
|  | Female | 28(52.83%) | 25(47.17%) |  | |  |
| Drinking | Yes | 77(44%) | 98(56%) | 2.14 | | 0.144 |
|  | No | 17(58.62%) | 12(41.38%) |  | |  |
| Smoking | Yes | 76(44.97%) | 93(55.03%) | 0.49 | | 0.485 |
|  | No | 18(51.43%) | 17(48.57%) |  | |  |
| Spleen thickness(mm) | ≤40 | 64(43.84%) | 82(56.16%) | 1.04 | | 0.308 |
|  | >40 | 30(51.72%) | 28(48.28%) |  | |  |
| Spleen length(mm) | ≤130 | 73(44.24%) | 92(55.76%) | 1.17 | | 0.279 |
|  | >130 | 21(53.85%) | 18(46.15%) |  | |  |
| BMI | 18～26 | 78(46.43%) | 90(53.57%) |  | | 0.926 |
|  | ＜18 | 1(50%) | 1(50%) |  | |  |
|  | ＞26 | 15(44.12%) | 19(55.88%) |  | |  |
| WBC (L^−1^, ×10⁹) | 3.5～9.5 | 80(44.69%) | 99(55.31%) |  | | 0.527 |
|  | ＞9.5 | 2(66.67%) | 1(33.33%) |  | |  |
|  | ＜3.5 | 12(54.55%) | 10(45.45%) |  | |  |
| HGB(g·L^−1^) | Male＜120; Female＜110 | 90(46.15%) | 105(53.85%) |  | | 1 |
|  |  | 4(44.44%) | 5(55.56%) |  | |  |
| RBC (L^−1^, ×10¹²) | Male≥4; Female≥3.5 | 84(44.44%) | 105(55.56%) | 2.76 | | 0.097 |
|  | Male＜4; Female＜3.5 | 10(66.67%) | 5(33.33%) |  | |  |
| PLT (L^−1^, ×10⁹) | ≥100 | 70(45.16%) | 85(54.84%) | 0.22 | | 0.640 |
|  | ＜100 | 24(48.98%) | 25(51.02%) |  | |  |
| N(L^−1^, ×10⁹) | 40～75 | 87(45.55%) | 104(54.45%) |  | | 0.540 |
|  | ＜40 | 4(44.44%) | 5(55.56%) |  | |  |
|  | ＞75 | 3(75%) | 1(25%) |  | |  |
| MON (L^−1^, ×10⁹) | ≤10 | 84(45.65%) | 100(54.35%) | 0.14 | | 0.711 |
|  | ＞10 | 10(50%) | 10(50%) |  | |  |
| EOS (L^−1^, ×10⁹) | ≤8 | 91(45.96%) | 107(54.04%) |  | | 1 |
|  | ＞8 | 3(50%) | 3(50%) |  | |  |
| PT(s) | ≤15 | 82(45.3%) | 99(54.7%) | 0.39 | | 0.534 |
|  | ＞15 | 12(52.17%) | 11(47.83%) |  | |  |
| APTT(s) | ≤35 | 53(44.92%) | 65(55.08%) | 0.15 | | 0.696 |
|  | ＞35 | 41(47.67%) | 45(52.33%) |  | |  |
| TT(s) | ≤18 | 33(42.86%) | 44(57.14%) | 0.52 | | 0.472 |
|  | ＞18 | 61(48.03%) | 66(51.97%) |  | |  |
| FIB(g/L) | ≥2 | 70(48.61%) | 74(51.39%) | 1.26 | | 0.261 |
|  | ＜2 | 24(40%) | 36(60%) |  | |  |
| ALT(U·L^−1^) | ≤40 | 41(53.25%) | 36(46.75%) | 2.56 | | 0.110 |
|  | ＞40 | 53(41.73%) | 74(58.27%) |  | |  |
| AST(U·L^−1^) | ≤40 | 42(50.6%) | 41(49.4%) | 1.15 | | 0.283 |
|  | ＞40 | 52(42.98%) | 69(57.02%) |  | |  |
| ALP(U·L^−1^) | ≤125 | 77(44.51%) | 96(55.49%) | 1.13 | | 0.288 |
|  | ＞125 | 17(54.84%) | 14(45.16%) |  | |  |
| GGT(U·L^−1^) | ≤60 | 63(48.09%) | 68(51.91%) | 0.6 | | 0.440 |
|  | ＞60 | 31(42.47%) | 42(57.53%) |  | |  |
| TBIL(μmol·L^−1^) | ＜17.1 | 56(43.75%) | 72(56.25%) | 0.75 | | 0.687 |
|  | 17.1～34.2 | 31(50%) | 31(50%) |  | |  |
|  | ＞34.2 | 7(50%) | 7(50%) |  | |  |
| GLO(g·L^−1^) | 20～40 | 87(44.85%) | 107(55.15%) |  | | 0.216 |
|  | ＜20 | 1(50%) | 1(50%) |  | |  |
|  | ＞40 | 6(75%) | 2(25%) |  | |  |
| ALB(g·L^−1^) | ≥40 | 63(46.32%) | 73(53.68%) | 0.01 | | 0.921 |
|  | ＜40 | 31(45.59%) | 37(54.41%) |  | |  |
| CR(μmol·L^−1^) | Male≤115;Female≤97 | 63(46.32%) | 73(53.68%) | 0.01 | | 0.921 |
|  | Male＞115; Female＞97 | 31(45.59%) | 37(54.41%) |  | |  |
| BUN(mmol·L^−1^) | 3.2～7.1 | 81(45.51%) | 97(54.49%) |  | | 0.681 |
|  | ＜3.2 | 10(55.56%) | 8(44.44%) |  | |  |
|  | ＞7.1 | 3(37.5%) | 5(62.5%) |  | |  |
| AFP(ng·mL^−1^) | ≤25 | 77(46.95%) | 87(53.05%) | 0.26 | | 0.613 |
|  | ＞25 | 17(42.5%) | 23(57.5%) |  | | . |
| HBeAg | positive | 76(44.97%) | 93(55.03%) | 0.49 | | 0.485 |
|  | negative | 18(51.43%) | 17(48.57%) |  | |  |
| HBeAb | positive | 76(44.97%) | 93(55.03%) | 0.49 | | 0.485 |
|  | negative | 18(51.43%) | 17(48.57%) |  | |  |
| inflammation grade | 3-6 | 34(55.74%) | 27(44.26%) | 3.41 | | 0.182 |
|  | 7-10 | 54(42.52%) | 73(57.48%) |  | |  |
|  | 11-14 | 6(37.5%) | 10(62.5%) |  | |  |
| Fibrosis | Ishak 5 | 38(49.35%) | 39(50.65%) | 0.53 | | 0.465 |
|  | Ishak 6 | 56(44.09%) | 71(55.91%) |  | |  |
| HBV DNA(Log_10_IU·mol^−1^) |  | 5.35(4.53, 6.83) | 7.15(6.02,8.00) | 0.53 | | 0.465 |

**Supplementary Table 4 Multivariate Logistic Regression Analysis of Factors Associated with Fibrosis Reversal in CHB Patients Treated with Entecavir Plus Biejia-Ruangan**

| Factor | Category Level | b | SE | OR(95% CI) | X^2^ | P value |
| --- | --- | --- | --- | --- | --- | --- |
| Fibrosis | Ishak 5 | Reference | | 1 |  |  |
|  | Ishak 6 | 0.373 | 0.314 | 1.452(0.785,2.687) | 1.413 | 0.235 |
| AGE | <45 | Reference |  | 1 |  |  |
|  | ≥45 | -0.427 | 0.300 | 0.652(0.362,1.175) | 2.023 | 0.155 |
| RBC(L^−1^, ×10⁹) | Male≥4; Female≥3.5 | Reference |  | 1 |  |  |
|  | Male＜4; Female＜3.5 | -1.156 | 0.608 | 0.315(0.096,1.036) | 3.6142 | 0.0573 |
| inflammation grade | 3-6 | Reference |  | 1 |  |  |
|  | 7-10 | 0.466 | 0.331 | 1.594(0.833,3.052) | 1.981 | 0.159 |
|  | 11-14 | 0.663 | 0.639 | 1.941(0.554,6.794) | 1.076 | 0.300 |
| ALT(U·L^−1^) | ≥40 | Reference |  | 1 |  |  |
|  | ＜40 | 0.304 | 0.315 | 1.356(0.732,2.512) | 0.937 | 0.333 |
| Dirnking | YES | Reference |  | 1 |  |  |
|  | NO | -0.483 | 0.426 | 0.617(0.268,1.422) | 1.286 | 0.257 |
| HBVDNA(Log_10_IU·mol) |  | 0.140 | 0.100 | 1.151(0.945,1.401) | 1.955 | 0.162 |
